# Optimising Multimodal Image Registration Techniques: A Comprehensive Study of Non-Rigid and Affine Methods for PET/CT Integration

**DOI:** 10.3390/diagnostics15192484

**Published:** 2025-09-28

**Authors:** Babar Ali, Mansour M. Alqahtani, Essam M. Alkhybari, Ali H. D. Alshehri, Mohammad Sayed, Tamoor Ali

**Affiliations:** 1 University Institute of Radiological Sciences and Medical Imaging Technology, The University of Lahore, Lahore 54000, Pakistan; babarali0741@gmail.com; 2 Department of Radiological Sciences, College of Applied Medical Sciences, Najran University, Najran 61441, Saudi Arabia; ahzafer@nu.edu.sa (A.H.D.A.); mfsayed@nu.edu.sa (M.S.); 3 Department of Radiology and Medical Imaging, College of Applied Medical Sciences, Prince Sattam Bin Abdulaziz University, P.O. Box 422, Alkharj 11942, Saudi Arabia; e.alkhybari@psau.edu.sa; 4 Department of Research, Government College University Lahore, Lahore 54000, Pakistan; alitamoor79@yahoo.com

**Keywords:** multimodal image registration, PET/CT integration, Demons algorithm, free, form deformation, preprocessing techniques, medical imaging optimisation

## Abstract

**Background/Objective**: Multimodal image registration plays a critical role in modern medical imaging, enabling the integration of complementary modalities such as positron emission tomography (PET) and computed tomography (CT). This study compares the performance of three widely used image registration techniques—Demons Image Registration with Modality Transformation, Free-Form Deformation using the Medical Image Registration Toolbox (MIRT), and MATLAB Intensity-Based Registration—in terms of improving PET/CT image alignment. **Methods**: A total of 100 matched PET/CT image slices from a clinical scanner were analysed. Preprocessing techniques, including histogram equalisation and contrast enhancement (via imadjust and adapthisteq), were applied to minimise intensity discrepancies. Each registration method was evaluated under varying parameter conditions with regard to sigma fluid (range 4–8), histogram bins (100 to 256), and interpolation methods (linear and cubic). Performance was assessed using quantitative metrics: root mean square error (RMSE), mean squared error (MSE), mean absolute error (MAE), the Pearson correlation coefficient (PCC), and standard deviation (STD). **Results**: Demons registration achieved optimal performance at a sigma fluid value of 6, with an RMSE of 0.1529, and demonstrated superior computational efficiency. The MIRT showed better adaptability to complex anatomical deformations, with an RMSE of 0.1725. MATLAB Intensity-Based Registration, when combined with contrast enhancement, yielded the highest accuracy (RMSE = 0.1317 at alpha = 6). Preprocessing improved registration accuracy, reducing the RMSE by up to 16%. **Conclusions**: Each registration technique has distinct advantages: the Demons algorithm is ideal for time-sensitive tasks, the MIRT is suited to precision-driven applications, and MATLAB-based methods offer flexible processing for large datasets. This study provides a foundational framework for optimising PET/CT image registration in both research and clinical environments.

## 1. Introduction

Multimodal medical imaging is the integration of two or more imaging modalities during a single examination, a process with both diagnostic and therapeutic implications. It provides comprehensive insights into the anatomical and functional characteristics of human tissues and has revolutionised diagnostic and treatment procedures [1,2,3]. The combination of positron emission tomography (PET) and computed tomography (CT)—known as PET/CT, a technique combining PET’s functional imaging capabilities with CT’s high-resolution anatomical details—has emerged as the gold standard in clinical and research contexts [4,5]. This synergistic combination permits precise localisation of metabolic disorders, boosting diagnostic accuracy in oncological, neurological, and cardiovascular applications [6]. The PET/CT imaging technique has transformed nuclear medicine through enhancing diagnostic accuracy, presenting clinical applicability, and offering personalised treatment strategies [7,8]. However, the correct fusion of PET and CT datasets necessitates reliable and precise image registration techniques, which align the images spatially to ensure coherence across modalities [9,10].

Medical image registration, defined as the process of aligning datasets such that they form a single coordinate system for multimodality imaging, is crucial. This approach is used to determine an optimal spatial transformation that aligns the underlying anatomical structures. Registration provides a deformation field that enables morphometric analysis, such as Jacobian-based volume-change mapping, in addition to cross-modal comparison [11]. It is utilised for a variety of clinical applications, including image guidance, motion tracking, segmentation, dose accumulation, and picture reconstruction [12]. Despite major breakthroughs, the intrinsic discrepancies between PET and CT—such as variances in spatial resolution, intensity profiles, and imaging artefacts—present substantial challenges for accurate registration. Traditional techniques, which frequently rely on rigid transformations, have proven inadequate for handling complicated deformations and mismatches in multimodal imaging. As a result, there has been increased emphasis on designing strong non-rigid and affine registration methods that can account for these complexities [11,13].

The Demons algorithm (also known as non-rigid registration) has received a lot of attention for its versatility and computing efficiency. It works on the basis of optical flow, using a fluid-like transformation model to align images. Despite its capabilities, the algorithm’s performance is influenced by several variables, including sigma fluid and interpolation methods [14,15,16]. Parameter tweaking is critical for improving accuracy, especially in cases involving substantial anatomical variability. Furthermore, the histogram bin sizes chosen in a given application have a significant impact on the algorithm’s capacity to capture intensity correlations between modalities, emphasising the importance of regular evaluations of these aspects [17]. Another popular image registration approach is free-form deformation (FFD), a technique used for efficient, smooth, and precise geometrical parametrisation; examples include the approaches implemented in the Medical Image Registration Toolbox (MIRT). In this technique, B-spline transformations are used to permit highly flexible deformations, making it ideal for correcting complex mismatches in multimodal datasets [18,19]. The MIRT has exhibited strong performance in a variety of therapeutic applications, particularly those requiring high precision. However, because of its processing requirements and sensitivity to parameter configurations such as sigma fluid values and histogram bins, careful optimisation is required to enhance its effectiveness [20,21]. Affine transformation is a geometric transformation, also known as an affinity, that is distinguished by its ability to preserve lines and parallelism but not necessarily Euclidean distances and angles. It is also commonly employed in medical imaging to correct for geometric imperfections. An example of this method is the MATLAB Intensity-Based Registration tool, which provides an efficient platform for performing affine transformations with user-defined parameters [22]. While affine approaches are less flexible than non-rigid techniques, their simplicity and computing efficiency make them desirable for some applications. Enhancements such as contrast stretching with functions like imadjust have been demonstrated to greatly enhance performance, especially with respect to datasets with difficult intensity distributions [23].

The incorporation of preprocessing techniques into registration processes has been recognised as a significant component of improving accuracy and dependability. Adapthisteq and imadjust have been found to efficiently eliminate intensity disparities and improve feature recognition [24]. These preprocessing processes are especially useful in multimodal imaging, where discrepancies in intensity profiles can conceal crucial features and prevent accurate registration [11,25]. Despite advances in registration algorithms and preparation techniques, there is still a paucity of complete comparative evaluations. Individual studies have shown the usefulness of several techniques in specific circumstances, but little is known about their relative performance across various parameter configurations and clinical scenarios [2,26,27,28]. This knowledge gap highlights the necessity of systematic evaluations that can help guide the selection and optimisation of registration procedures depending on application requirements.

In this study, we sought to address these gaps by conducting a comprehensive direct head-to-head comparison of three well-known registration techniques—Demons Image Registration with Modality Transformation, MIRT-based Free-Form Deformation, and MATLAB Intensity-Based Registration—in the context of PET/CT imaging. We aimed to identify the best configurations for every technique by carefully adjusting critical factors, including sigma fluid, histogram bins, and interpolation algorithms. Moreover, we investigated the impact of preprocessing processes, such as contrast enhancement and histogram equalisation, on registration performance. The findings are intended to provide useful insights into the relative strengths and limitations of various techniques, laying the groundwork for their use in both clinical and research settings.

## 2. Materials and Methods

### 2.1. Ethical Consideration, Dataset Acquisition, and Registration Techniques

The study was conducted in accordance with the Declaration of Helsinki [29] and received ethical approval from the Research Ethics Committee, Faculty of Allied Health Sciences, University of Lahore (Ref No: REC-UOL-501-08-2024), on 18 August 2024. Prior to participation, informed consent was obtained from all the patients, ensuring their voluntary involvement and awareness of the research procedures.

We used real-time PET/CT image datasets obtained from a clinical PET/CT scanner available at the Institute of Nuclear Medicine & Oncology Lahore (INMOL). A total of 100 thoracic PET/CT slices were obtained from both male and female patients aged 40–70. The figures were limited to thoracic transaxial slices to ensure consistency across methods. PET/CT studies were performed using a clinical PET/CT scanner (Siemens Biograph mCT, Siemens Healthineers, Erlangen, Germany). For PET acquisition, we used ^18^F-FDG (370 MBq, 60 min uptake), reconstructed with OSEM (4 iterations × 16 subsets, 5 mm Gaussian filter). CT scans were acquired at 120 kVp at 150 mAs, with a slice thickness of 5 mm and a matrix size of 512 × 512. All images were exported in DICOM format. The datasets were anonymised to protect patient confidentiality and ensure that biomedical research ethics criteria were followed. Images with severe motion or acquisition artefacts were excluded to ensure quality. Slice selection and quality assessment were performed independently by two board-certified radiologists, with ground-truth correspondence for this dataset established through their consensus review of PET/CT alignment. This approach is consistent with strategies reported in prior multimodal registration studies, which also emphasised radiologist consensus as the most reliable standard for establishing image truth and minimising artefacts [11,12]. Processing was performed on a Dell Precision 5820 workstation (Intel Xeon Silver, 64 GB of RAM, Windows 10). MATLAB 2012a was selected to ensure compatibility with the MIRT and maintain reproducibility in clinical environments wherein legacy systems are still in use [30].

**Figure 1:**
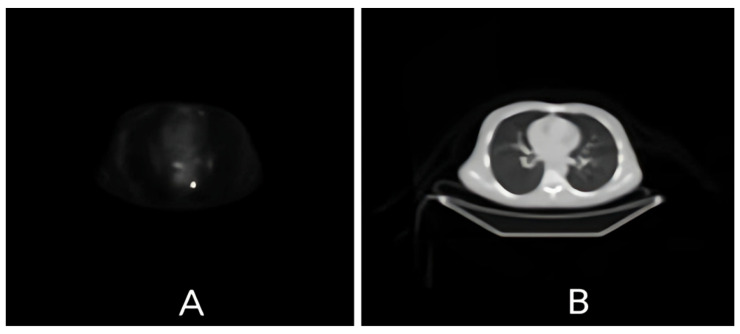
Real-time PET (**A**) and CT (**B**) images acquired from the same patient dataset. PET provides functional metabolic information, whereas CT offers high-resolution anatomical detail. These paired images were used as inputs for subsequent registration experiments.

The PET images yielded functional data on metabolic activity, while the CT images included high-resolution anatomical details, as seen in Figure 1, resulting in a multimodal dataset suitable for registration evaluation. In order to register these real-time images, three different methods were used: Demons image registration with modality transformation [31], the MIRT using free-form deformation [32], and the following MATLAB 2012a toolbox: intensity-based multimodality image registration of image processing [30]. The same slices of PET and CT images were used in each method.

**Figure 2:**
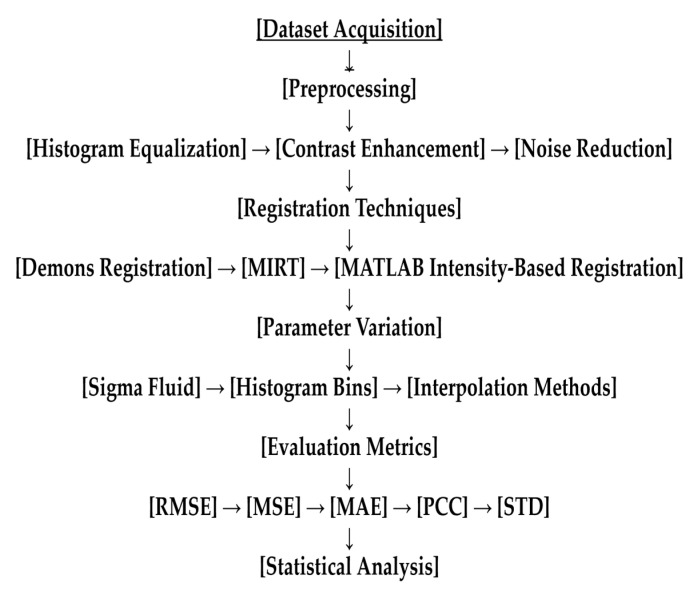
Experimental workflow diagram. The process included preprocessing steps (NR = noise reduction; HE = histogram equalisation; and CE = contrast enhancement), registration methods (Demons, MIRT, and MATLAB), and evaluation measures (RMSE = root mean square error, MSE = mean square error, MAE = mean absolute error, PCC = Pearson correlation coefficient, and STD = standard deviation).

We assessed the Demons algorithm, the MIRT, and MATLAB Intensity-Based Registration tools in terms of their ability to capture complex deformations and align multimodal images. We compared parameter configurations, sigma fluid values, and interpolation approaches, with an emphasis on high-variance datasets and processing performance. To improve accuracy, we also tested alpha parameters, histogram configurations, and linear and cubic interpolation approaches. A complete experimental workflow diagram is provided in Figure 2.

### 2.2. Preprocessing Techniques

Prior to registration, PET/CT images were pre-processed to improve their quality and interpretability. The key steps were as follows:

- Histogram Equalisation: The adapthisteq function was used to standardise intensity distributions across modalities.
- Contrast Enhancement: The imadjust function was employed to improve visibility and feature identification, especially in low-contrast environments.
- Noise Reduction: Filters were used to reduce noise artefacts and provide cleaner inputs for registration.

Preprocessing has been emphasised as a crucial step in multimodal registration, with recent reviews highlighting its impact on reproducibility and radiomic feature stability [24].

### 2.3. Evaluation Metrics

We study used a set of parameters to quantitatively evaluate registration performance:

- Root Mean Square Error (RMSE): This metric is used to calculate the average difference between corresponding pixel intensities in registered pictures.
- Mean Square Error (MSE): This metric quantifies squared differences, highlighting larger misalignments.
- Mean Absolute Error (MAE): This metric is used to calculate absolute differences and provides a direct assessment of accuracy.
- Pearson Correlation Coefficient (PCC): This metric measures the linear correlation between PET and CT images after registration.
- Standard Deviation (STD): This metric is used to measure intensity variability to assess registration consistency.

These metrics are consistent with widely used evaluation frameworks in the PET/CT registration literature [26,27,28].

### 2.4. Parameter Variations

Parameter ranges were set based on prior reports and empirical evidence. Sigma fluid values between 4 and 8 are commonly used to balance smoothness and deformation accuracy. Histogram bin sizes from 100 to 256 enabled evaluation of both fine-grained and coarse intensity mappings. Alpha values (4–8) were tested in MATLAB-based affine registration to explore different levels of transformation flexibility.

#### 2.4.1. Sigma Fluid and Alpha Values

The sigma fluid values for the Demons and MIRT techniques were changed from 4 to 8 to see how they affected registration accuracy. Similarly, alpha values in the MATLAB-based programme were changed to maximise affine transformations.

#### 2.4.2. Histogram Bin Sizes

The effects of histogram bin widths ranging from 100 to 256 were investigated. Smaller bins were expected to improve alignment accuracy by yielding finer-intensity mappings, whereas larger bins were expected to smooth intensity correlations.

#### 2.4.3. Interpolation Methods

Linear and cubic interpolation methods were examined for all strategies. Linear interpolation was expected to be computationally efficient, while cubic interpolation was thought to improve accuracy at the expense of processing time.

### 2.5. Statistical Analysis

Registration performance was evaluated quantitatively using five metrics: RMSE, MSE, MAE, PCC, and STD. For each parameter configuration and registration method, values were calculated across 100 PET/CT slices and summarised as means ± standard deviations. The results were compared descriptively to identify trends in accuracy and consistency across sigma fluid values, histogram bin sizes, alpha values, and interpolation methods. Improvements were interpreted based on relative changes in mean values, with particular attention paid to the trade-off between accuracy and variability. No formal hypothesis testing was performed; instead, the analysis emphasised practical differences directly relevant to clinical applications.

## 3. Results

### 3.1. Demons Image Registration

**Figure 3:**
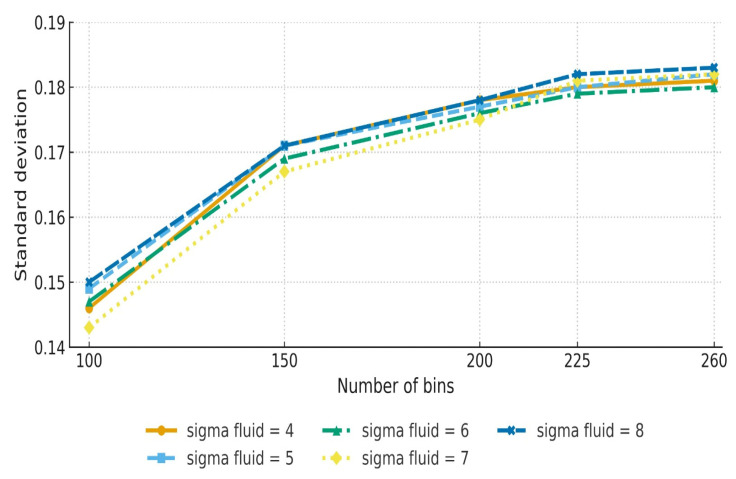
The STD of registration performance across different sigma fluid values and histogram bin sizes (*n* = 100 PET/CT slice pairs). The *x*-axis represents histogram bin size (number of bins), and the *y*-axis represents standard deviation values. Higher STDs indicate greater variability in alignment, with a sigma = 6 showing more stable performance across parameter ranges.

**Figure 4:**
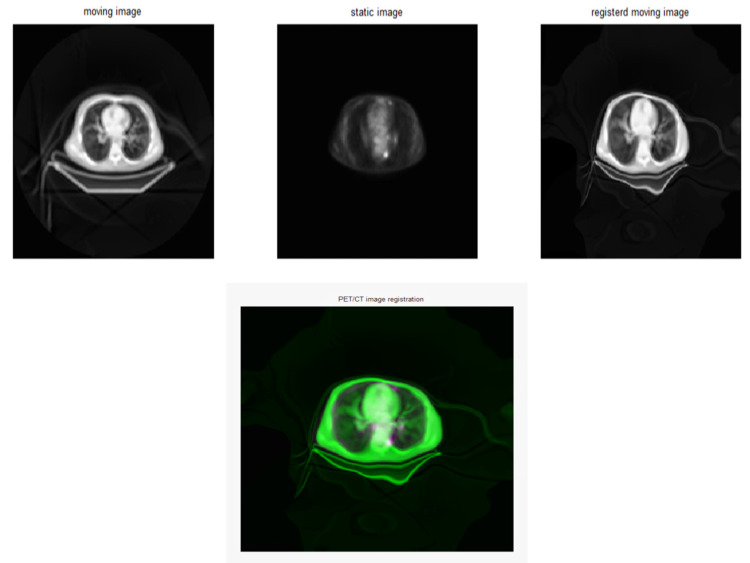
Example of Demons registration from LHS, with CT corresponding to the moving image and with PET as the static reference (n = 1 case shown) and registered moving image, when using a sigma fluid value = 4, a no. of bins = 256, and the contrast-stretching function adapthisteq. CT was selected as the fixed modality because of its superior spatial resolution, while PET was changed to CT to preserve anatomical accuracy.

The effect of changing the sigma fluid values from 4 to 8 was examined. The investigation showed that higher sigma fluid values resulted in modest improvements in RMSE and MSE. The best outcome was obtained at a sigma fluid level = 6, indicating significant alignment precision. As sigma fluid levels rose over this threshold, measurements such as MAE and PCC showed a minor decline (Table 1, Figure 3). Figure 4 shows the effect of Demons registration when using a sigma fluid value = 4; 256 bins; and the contrast-stretching function adapthisteq.

**Figure 5:**
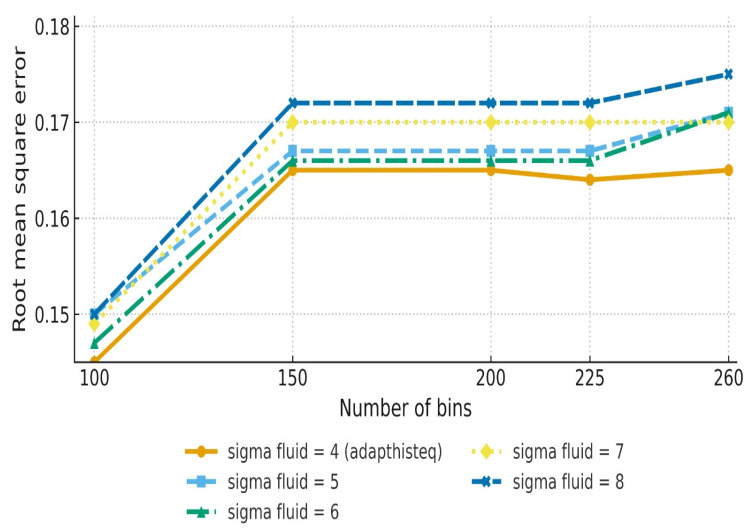
RMSE plotted against histogram bin sizes for different sigma fluid values using adapthisteq preprocessing (n = 100 PET/CT slice pairs). The error bars indicate the standard deviation across slices. The *x*-axis represents histogram bin size, and the *y*-axis represents RMSE. Lower bin counts yielded sharper alignment but less consistency across patients.

Histogram bin sizes ranging from 100 to 256 were investigated to determine their effect on registration quality. Our investigation revealed that smaller bins sizes (for example, 100 bins) produced crisper and more accurate registration results. However, increasing bin sizes caused modest smoothing artefacts, especially in high-gradient zones (Table 2, Figure 5). Although Table 2 shows that the lowest RMSE occurred with a sigma fluid value = 4 at 100 bins, sigma = 6 was considered optimal because it provided balanced performance across RMSE, MAE, PCC, and stability. The designation of sigma = 6 as “optimal” reflects balanced outcomes rather than optimisation of a single metric, as reported in Table 1.

We assessed linear and cubic interpolation algorithms. Linear interpolation consistently surpassed cubic interpolation in all criteria, providing a good compromise between computational efficiency and registration accuracy. Linear interpolation outperformed cubic interpolation because cubic smoothing reduced edge sharpness in high-gradient PET tumour regions, leading to less-accurate alignment. Linear interpolation preserved these edges, resulting in superior accuracy despite its relative simplicity (Table 3).

### 3.2. MIRT with Free-Form Deformation

The MIRT demonstrated excellent adaptation to altering sigma fluid conditions. Sigma fluid = 6 led to the best performance, with minimal RMSE and MSE values, ensuring accurate non-rigid deformations (Table 4).

**Figure 6:**
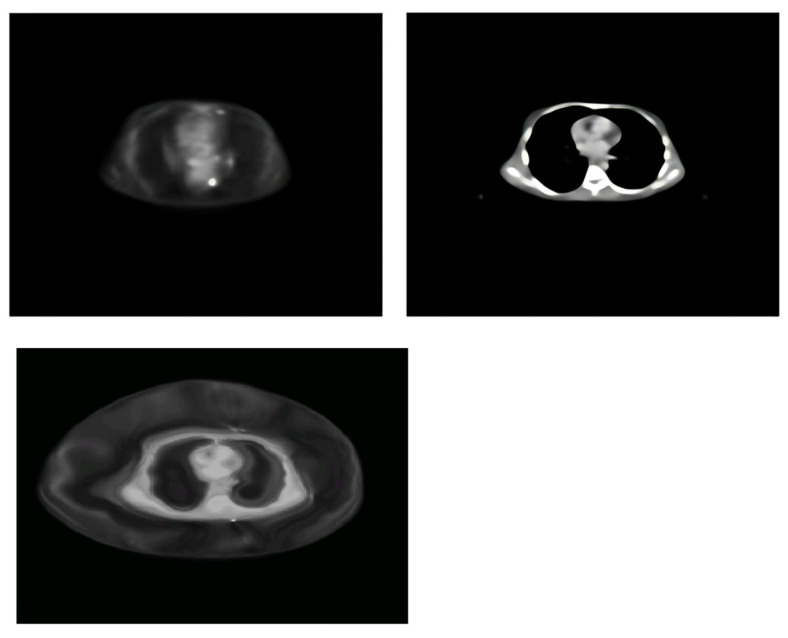
Example of MIRT registration. From LHS: PET images were used as a source, and CT images were used as a reference; the result of registration when using 100 bins is shown. This configuration provided improved alignment of subtle anatomical structures.

Smaller bins enhanced registration accuracy, especially in areas with slight intensity variations. Larger bins resulted in mild smoothing effects that reduced precision (Table 5). The effect on MIRT registration performance when using 100 bins is shown in Figure 6.

### 3.3. MATLAB Intensity-Based Registration

**Figure 7:**
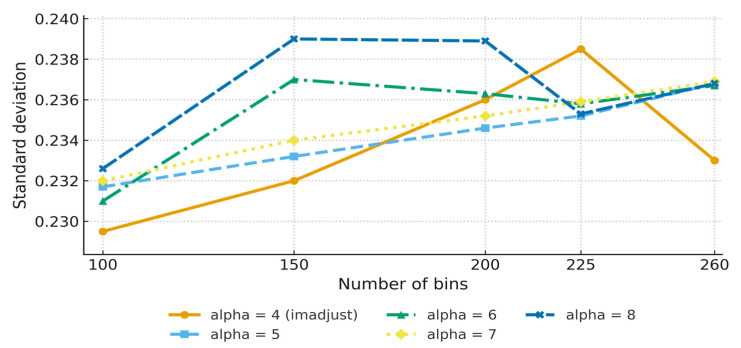
Relationship between no. of bins and STD with different alpha values when using imadjust function preprocessing (n = 100 slices). Error bars represent variability across slices. The *x*-axis shows the number of bins, and the *y*-axis shows STD values. Although lower alpha values produced slightly better RMSEs, alpha = 6 yield more consistent overall performance.

**Figure 8:**
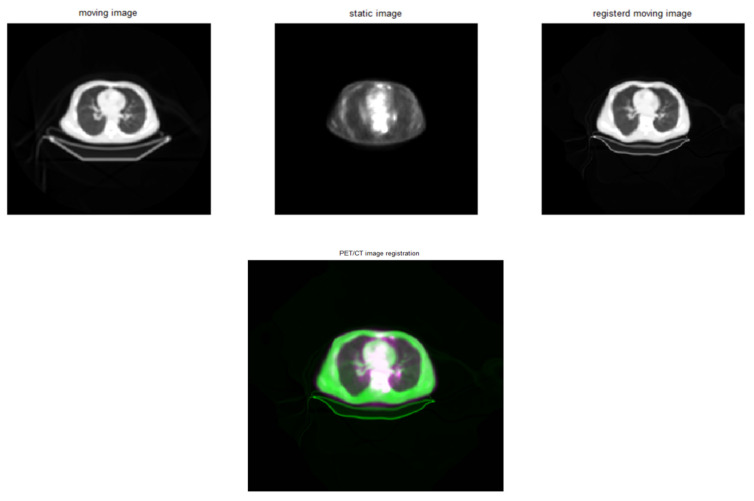
MATLAB intensity-based registration results, with CT as a moving image, PET as a static image, alpha = 4, 256 bins, and imadjust preprocessing. CT was changed to PET to evaluate affine registration flexibility, though PET-to-CT alignment remains clinically preferable in most cases.

The effect of alpha values on registration accuracy was studied. Alpha = 6 was identified as the best option, leading to balanced precision across all measurements (Table 6, Figure 7). Figure 8 shows the effect of MATLAB intensity-based registration when using an alpha = 4; 256 bins; and the contrast-stretching function imadjust. While RMSE values were lowest at alpha = 4 and 5, alpha = 6 produced more stable outcomes across all accuracy metrics and demonstrated lower variability in repeated trials. This stability justified selecting alpha = 6 as the most balanced configuration (Table 6).

Contrast enhancement using the imadjust function improved registration performance dramatically, particularly in regard to low-contrast PET/CT datasets. Contrast stretching reduced RMSEs and improved alignment but simultaneously increased STDs, indicating greater variability in intensity values. This trade-off suggests that preprocessing improves correspondence but can also amplify differences in noisy regions (Table 7).

## 4. Discussion

Multimodal image registration remains a cornerstone of medical imaging, with PET/CT integration being particularly important for clinical diagnoses and research. This study evaluates three popular registration techniques—Demons Image Registration, MIRT-Based Free-Form Deformation, and MATLAB Intensity-Based Registration—across a range of parameter settings, preprocessing approaches, and assessment criteria.

The Demons algorithm demonstrated a solid balance between computational efficiency and registration accuracy, consistent with previous findings that highlighted its suitability for clinical applications requiring both speed and reliability. Table 1 shows that this approach performed optimally for a sigma fluid value = 6, with an RMSE of 0.1529 and an MAE of 0.0543. Histogram bin selection was also important, with lower bin sizes, such as 100, producing higher accuracy metrics than larger bins. This shows Demons registration’s sensitivity to intensity granularity. However, this approach encountered difficulties when dealing with complex non-rigid deformations, which could be an important factor for clinical situations featuring considerable anatomical variability. Recent studies confirm this observation, noting that Demons registration performs well in simpler anatomical regions but struggles with high non-rigid deformation, such as respiratory or cardiac motion [16].

The MIRT’s ability to capture complex anatomical deformations was demonstrated by its constant performance across multiple parameter settings. The MIRT demonstrated resilience, with an RMSE of 0.1725 and a PCC of 4.0008 × 10^4^. However, its computational needs were significantly higher, with processing times almost 1.5 times longer than those of the Demons algorithm. This trade-off between precision and efficiency makes the MIRT an excellent choice for research-oriented applications that require high accuracy, but less so for time-critical clinical processes. These findings are consistent with prior work reporting that free-form deformation approaches, while computationally intensive, facilitate superior alignment for organs subject to complex deformation, such as the liver and thoracic structures [27,28].

The MATLAB-based technique stood out because of its simplicity and adaptability, aligning with earlier studies that emphasised the robustness of affine registration strategies in hybrid workflows and their utility in diverse clinical datasets [23]. The use of preprocessing techniques, particularly contrast augmentation with imadjust, led to much better registration results. At an alpha = 6, this approach had an RMSE of 0.1317 and an MAE of 0.0402, demonstrating its usefulness for datasets with complex intensity profiles. Affine registration strategies, although less flexible, continue to be applied in hybrid workflows due to their robustness and simplicity, particularly when combined with intensity-based preprocessing [18,23]. A comparison of linear and cubic interpolation methods found that the former regularly outperformed the latter in terms of RMSE and MAE, as shown in Table 6.

Preprocessing approaches have emerged as a key factor in registration performance. Histogram equalisation and contrast enhancement effectively mitigated intensity disparities, especially in low-contrast PET/CT datasets, lowering the RMSE by as much as 16%, as evidenced by comparisons using imadjust and adapthisteq, demonstrating the importance of integrated preprocessing pipelines in multimodal registration workflows.

Clinically, the Demons algorithm demonstrated a strong balance between computational efficiency and registration accuracy, making it well suited for time-sensitive applications such as adaptive radiotherapy, where fast yet reliable performance is essential. In contrast, the MIRT-based free-form deformation approach, although more computationally intensive, has been shown to allow superior alignment in anatomically complex regions such as the thorax and abdomen, supporting its utility in high-precision applications like neuroimaging research [27]. The MATLAB-based affine registration technique stood out in this study for its simplicity and adaptability, particularly when combined with preprocessing methods, a finding consistent with previous reports highlighting the robustness of affine strategies in multimodal registration tasks [23]. By comparison, deep-learning-based methods such as VoxelMorph and Quicksilver offer excellent accuracy and speed but require large, annotated datasets and extensive computational resources and often lack transparency, which limits their current clinical translation [33,34]. Beyond these approaches, recent work has extended affine and non-rigid registration methods using deep learning. Chen et al. (2025) provided a comprehensive survey of new technologies, uncertainty modelling, and evaluation metrics in deep learning registration, underscoring both progress and ongoing challenges [35]. Similarly, Trotter et al. (2023) and Hussain et al. (2024) emphasised deep learning’s increasing role in PET/CT imaging for segmentation, enhancement, and registration-related applications while also noting persistent barriers such as data scarcity, computational demands, and limited interpretability [1,5]. Taken together, these findings highlight that while deep-learning approaches continue to advance rapidly, classical registration algorithms remain highly relevant in clinical workflows because of their interpretability, reproducibility, and practical feasibility.

Several commercial PET/CT registration and visualisation platforms are available, including Mirada XD (Mirada Medical, https://mirada-medical.com), Hermia (Hermes Medical Solutions, https://www.hermesmedical.com), AIDAN (Siemens Healthineers, https://www.siemens-healthineers.com), and Medicalholodeck (https://www.medicalholodeck.com). Open-source options such as 3D Slicer (https://www.slicer.org) and AMIDE (http://amide.sourceforge.net) are also widely used in clinical and research settings. Unlike these established tools, our approach focuses on transparent and reproducible MATLAB-based implementations of classical methods (Demons, MIRT, and affine), allowing detailed parameter tuning and benchmarking, thereby providing methodological insights that complement the functionality of commercial platforms, which often operate as closed systems.

This study has several limitations. The dataset was relatively small (100 slices from 15 oncologic patients), which may limit generalisability. Although 3D volumetric registration is standard in clinical practice, only 2D slices were analysed in this study; this choice was made to reduce computational demand and allow controlled parameter comparisons across algorithms. The dataset was also relatively homogeneous, consisting primarily of thoracic PET/CT slices, improving internal consistency but restricting applicability to other anatomical regions and more diverse patient populations. MATLAB 2012a was used to ensure compatibility with the employed toolboxes, although newer platforms may offer enhanced functionality. Additionally, the figures primarily illustrated thoracic slices, limiting the demonstration of anatomical variability. Furthermore, this study focused exclusively on technical registration performance and neglected diagnostic accuracy. Deep-learning-based registration was not directly implemented, though it was discussed as a future direction. Future investigations should incorporate larger and more heterogeneous datasets, extend analyses to 3D volumetric registration, and explore hybrid classical deep-learning approaches. Similar recommendations have been made in recent reviews by Darzi and Bocklitz [11] and Ramadan et al. [12]. Finally, as this study relied exclusively on institutional data, external validation using publicly available repositories such as The Cancer Imaging Archive (TCIA) [36] will be an important step to improve generalisability across populations and imaging platforms.

## 5. Conclusions and Clinical Implications

This comprehensive evaluation of multimodal image registration techniques highlights the unique strengths and limitations of Demons Image Registration, MIRT-Based Free-Form Deformation, and MATLAB Intensity-Based Registration. By systematically varying parameters such as sigma fluid, histogram bins, and interpolation methods, we identified optimal configurations for each technique, tailored to specific applications. Demon image registration demonstrated significant computational efficiency, exhibiting optimal performance at a sigma fluid value of 6, with an RMSE of 0.1529. The MIRT was effective at managing complicated anatomical deformations, achieving an RMSE of 0.1725 at a sigma fluid = 6, albeit with higher computational demands, whereas MATLAB-based image registration improved with preprocessing approaches such as contrast enhancement, achieving an RMSE of 0.1317 at an alpha = 6 and highlighting its adaptability regarding datasets with challenging intensity profiles.

This study’s findings have direct implications for clinical workflows. Demons registration, with its computational efficiency, is ideal for applications like radiation therapy planning, where speed is critical. In contrast, the MIRT’s outstanding precision makes it appropriate for applications that require precise deformations, such as neuroimaging research. MATLAB-based image registration is a versatile option, especially when preprocessing is incorporated into the workflow to handle datasets with complex intensity distributions. The findings of our research illustrate each technique’s strengths and weaknesses, emphasising the importance of parameter adjustment and preprocessing for achieving optimal outcomes. Ultimately, this study provides a core paradigm for optimising multimodal image registration algorithms, providing important insights for clinical and research applications. By addressing significant challenges in PET/CT integration, this study’s outcomes enhance diagnostic accuracy in medical imaging and contribute to advancing precision medicine. Future research studies should focus on hybrid approaches and machine learning-based optimisation techniques to improve the precision and efficiency of multimodality image registration.

## Figures and Tables

**Table 1:** Demons registration performance metrics for varying sigma fluid values.

Sigma Fluid	RMSE	MSE	MAE	PCC	STD
4	0.1667	0.0278	0.0626	4.0035 × 10^4^	0.1787
5	0.1612	0.0259	0.0598	4.0500 × 10^4^	0.1734
6 (optimal)	0.1529	0.0234	0.0543	4.0785 × 10^4^	0.1676
7	0.1635	0.0270	0.0610	4.0256 × 10^4^	0.1762
8	0.1748	0.0305	0.0713	3.9627 × 10^4^	0.1835

**Table 2:** Demons registration performance metrics for varying histogram bins.

Bins	RMSE	MSE	MAE	PCC	STD
100	0.1260	0.0159	0.0373	3.8451 × 10^4^	0.1434
155	0.1515	0.0229	0.0522	4.1803 × 10^4^	0.1676
200	0.1605	0.0247	0.0578	4.1200 × 10^4^	0.1728
224	0.1648	0.0272	0.0623	4.0681 × 10^4^	0.1773
256	0.1700	0.0293	0.0650	4.0352 × 10^4^	0.1832

**Table 3:** Comparison of interpolation methods in regard to Demons registration.

Interpolation	RMSE	MSE	MAE	PCC	STD
Linear	0.1546	0.0278	0.0540	4.1904 × 10^4^	0.1704
Cubic	0.1753	0.0307	0.0699	4.0000 × 10^4^	0.1850

**Table 4:** MIRT performance metrics across sigma fluid values.

Sigma Fluid	RMSE	MSE	MAE	PCC	STD
4	0.1686	0.0284	0.0639	4.0527 × 10^4^	0.1806
5	0.1653	0.0276	0.0612	4.0102 × 10^4^	0.1771
6 (optimal)	0.1725	0.0298	0.0680	4.0008 × 10^4^	0.1817
7	0.1742	0.0302	0.0701	3.9750 × 10^4^	0.1843
8	0.1767	0.0312	0.0717	3.9639 × 10^4^	0.1854

**Table 5:** MIRT registration performance for varying histogram bins.

Bins	RMSE	MSE	MAE	PCC	STD
100	0.1312	0.0171	0.0400	3.8515 × 10^4^	0.1478
155	0.1722	0.0296	0.0679	4.0035 × 10^4^	0.1800

**Table 6:** Affine transformation metrics for MATLAB registration.

Alpha	RMSE	MSE	MAE	PCC	STD
4	0.1302	0.0170	0.0390	3.8698 × 10^4^	0.1475
5	0.1298	0.0168	0.0388	3.8750 × 10^4^	0.1463
6	0.1317	0.0173	0.0402	3.8615 × 10^4^	0.1485
7	0.1325	0.0175	0.0408	3.8550 × 10^4^	0.1501
8	0.1307	0.0171	0.0400	3.8700 × 10^4^	0.1476

**Table 7:** Impact of contrast enhancement on registration accuracy.

Contrast Stretch	RMSE	MSE	MAE	PCC	STD
Without	0.1529	0.0234	0.0543	4.0785 × 10^4^	0.1676
With	0.1285	0.0165	0.0480	4.3452 × 10^4^	0.2308

## Data Availability

The datasets analysed during the current study are not publicly available owing to institutional ethical restrictions but can be obtained from the corresponding author upon reasonable request.

## Abbreviations

The following abbreviations are used in this manuscript:

PET	Positron Emission Tomography
CT	Computed Tomography
FDG	Fluorodeoxyglucose (^18^F)
ROI	Region of Interest
RMSE	Root Mean Square Error
MSE	Mean Square Error
MAE	Mean Absolute Error
PCC	Pearson Correlation Coefficient
STD	Standard Deviation
FFD	Free-Form Deformation
CE	Contrast Enhancement
HE	Histogram Equalisation
OSEM	Ordered-Subset Expectation Maximisation
MIRT	Michigan Image Reconstruction Toolbox

## References

[1] Hussain D., Abbas N., Khan J. (2024). Recent Breakthroughs in PET-CT Multimodality Imaging: Innovations and Clinical Impact. Bioengineering.

[2] Zubair M., Hussain M., Albashrawi M.A., Bendechache M., Owais M. (2025). A comprehensive review of techniques, algorithms, advancements, challenges, and clinical applications of multi-modal medical image fusion for improved diagnosis. Comput. Methods Programs Biomed..

[3] Haribabu M., Guruviah V., Yogarajah P. (2023). Recent Advancements in Multimodal Medical Image Fusion Techniques for Better Diagnosis: An Overview. Curr. Med. Imaging.

[4] Sun Y., Cheng Z., Qiu J., Lu W. (2024). Performance and application of the total-body PET/CT scanner: A literature review. EJNMMI Res..

[5] Trotter J., Pantel A.R., Teo B.K., Escorcia F.E., Li T., Pryma D.A., Taunk N.K. (2023). Positron Emission Tomography (PET)/Computed Tomography (CT) Imaging in Radiation Therapy Treatment Planning: A Review of PET Imaging Tracers and Methods to Incorporate PET/CT. Adv. Radiat. Oncol..

[6] Nogueira-Lima E., Alves T., Etchebehere E. (2024). 18F-Fluoride PET/CT-Updates. Semin. Nucl. Med..

[7] Lee J., Kim T. (2025). Current Status and Future Perspectives of Nuclear Medicine in Prostate Cancer from Imaging to Therapy: A Comprehensive Review. Biomedicines.

[8] Singh M.K. (2024). A review of digital PET-CT technology: Comparing performance parameters in SiPM integrated digital PET-CT systems. Radiography.

[9] Aide N., Lasnon C., Desmonts C., Armstrong I.S., Walker M.D., McGowan D.R. (2022). Advances in PET/CT Technology: An Update. Semin. Nucl. Med..

[10] Khalil A., Ng S.C., Liew Y.M., Lai K.W. (2018). An Overview on Image Registration Techniques for Cardiac Diagnosis and Treatment. Cardiol. Res. Pract..

[11] Darzi F., Bocklitz T. (2024). A Review of Medical Image Registration for Different Modalities. Bioengineering.

[12] Ramadan H., El Bourakadi D., Yahyaouy A., Tairi H. (2024). Medical image registration in the era of Transformers: A recent review. Inform. Med. Unlocked.

[13] Shiyam Sundar L.K., Lassen M.L., Gutschmayer S., Ferrara D., Calabrò A., Yu J., Kluge K., Wang Y., Nardo L., Hasbak P. (2023). Fully Automated, Fast Motion Correction of Dynamic Whole-Body and Total-Body PET/CT Imaging Studies. J. Nucl. Med..

[14] Wang R., Zhang B. (2024). Improved Demons algorithm for non-rigid medical image alignment. Applied Mathematics and Nonlinear Sciences.

[15] Glira P., Weidinger C., Otepka-Schremmer J., Ressl C., Pfeifer N., Haberler-Weber M. (2023). Nonrigid Point Cloud Registration Using Piecewise Tricubic Polynomials as Transformation Model. Remote Sens..

[16] Roy A., Roy P.K., Mitra A., Daw S., Basu D., Chakraborty S. (2025). Revolutionizing nonrigid demons registration with the whale optimization algorithm. Int. J. Electr. Comput. Eng..

[17] Song G., Han J., Zhao Y., Wang Z., Du H. (2017). A Review on Medical Image Registration as an Optimization Problem. Curr. Med. Imaging Rev..

[18] Sun W., Niessen W.J., Klein S. (2014). Free-form deformation using lower-order B-spline for nonrigid image registration. Med. Image Comput. Comput. Assist. Interv..

[19] Shi W., Zhuang X., Pizarro L., Bai W., Wang H., Tung K.P., Edwards P., Rueckert D. (2012). Registration using sparse free-form deformations. Med. Image Comput. Comput. Assist. Interv..

[20] Afzali M., Ghaffari A., Fatemizadeh E., Soltanian-Zadeh H. (2016). Medical image registration using sparse coding of image patches. Comput. Biol. Med..

[21] Nie Q., Zhang X., Hu Y., Gong M., Liu J. (2024). Medical image registration and its application in retinal images: A review. Vis. Comput. Ind. Biomed. Art.

[22] Mandl L., Mielke A., Seyedpour S.M., Ricken T. (2023). Affine transformations accelerate the training of physics-informed neural networks of a one-dimensional consolidation problem. Sci. Rep..

[23] Negi S.S., Bhandari Y.S. A hybrid approach to image enhancement using contrast stretching on image sharpening and the analysis of various cases arising using histogram. Proceedings of the International Conference on Recent Advances and Innovations in Engineering (ICRAIE-2014).

[24] Trojani V., Bassi M.C., Verzellesi L., Bertolini M. (2024). Impact of Preprocessing Parameters in Medical Imaging-Based Radiomic Studies: A Systematic Review. Cancers.

[25] Iakab S.A., Ràfols P., Correig-Blanchar X., García-Altares M. (2021). Perspective on Multimodal Imaging Techniques Coupling Mass Spectrometry and Vibrational Spectroscopy: Picturing the Best of Both Worlds. Anal Chem..

[26] Ou Y., Akbari H., Bilello M., Da X., Davatzikos C. (2014). Comparative evaluation of registration algorithms in different brain databases with varying difficulty: Results and insights. IEEE Trans. Med. Imaging..

[27] Vogel W.V., van Dalen J.A., Wiering B., Huisman H., Corstens F.H., Ruers T.J., Oyen W.J. (2007). Evaluation of image registration in PET/CT of the liver and recommendations for optimized imaging. J. Nucl. Med..

[28] Mattes D., Haynor D.R., Vesselle H., Lewellen T.K., Eubank W. (2003). PET-CT image registration in the chest using free-form deformations. IEEE Trans. Med. Imaging.

[29] World Medical Association (2025). World Medical Association Declaration of Helsinki: Ethical Principles for Medical Research Involving Human Participants. JAMA.

[30] MathWorks (2012). MATLAB and Image Processing Toolbox Release 2012a.

[31] Gu X., Pan H., Liang Y., Castillo R., Yang D., Choi D., Castillo E., Majumdar A., Guerrero T., Jiang S.B. (2010). Implementation and evaluation of various demons deformable image registration algorithms on a GPU. Phys. Med. Biol..

[32] Fessler J.A. Michigan Image Reconstruction Toolbox (MIRT). University of Michigan. http://web.eecs.umich.edu/~fessler/code/.

[33] Balakrishnan G., Zhao A., Sabuncu M.R., Guttag J., Dalca A.V. (2019). VoxelMorph: A learning framework for deformable medical image registration. IEEE Trans. Med. Imaging.

[34] Yang X., Kwitt R., Styner M., Niethammer M. (2017). Quicksilver: Fast predictive image registration—A deep learning approach. Neuroimage.

[35] Chen J., Liu Y., Wei S., Bian Z., Subramanian S., Carass A., Prince J.L., Du Y. (2025). A survey on deep learning in medical image registration: New technologies, uncertainty, evaluation metrics, and beyond. Med. Image Anal..

[36] Clark K., Vendt B., Smith K., Freymann J., Kirby J., Koppel P., Moore S., Phillips S., Maffitt D., Pringle M. (2013). The Cancer Imaging Archive (TCIA): Maintaining and operating a public information repository. J. Digit. Imaging.

